# Association of Preoperative Plateletcrit and Other Inflammatory Indices with Postoperative Pain Severity and Opioid Consumption in Morbidly Obese Patients Receiving Transversus Abdominis Plane Block: A Retrospective Observational Study

**DOI:** 10.3390/metabo16100747

**Published:** 2026-10-04

**Authors:** Mesut Oterkus, Ümit Karatepe, Hacı Osman Toplu, Cihan Doger

**Affiliations:** 1 Department of Anesthesiology and Reanimation, Faculty of Medicine, Malatya Turgut Özal University, 44280 Malatya, Türkiye; mesut.oterkus@ozal.edu.tr; 2 Department of Anesthesiology and Reanimation, Elazığ Fethi Sekin City Hospital, 23280 Elazığ, Türkiye; 3 Department of Anesthesiology and Reanimation, Malatya Training and Research Hospital, 44000 Malatya, Türkiye; hosmantoplu@gmail.com; 4 Department of Anaesthesiology and Reanimation, Ankara Bilkent City Hospital, 06800 Ankara, Türkiye; cihandoger@gmail.com

**Keywords:** laparoscopic sleeve gastrectomy, TAP block, immunometabolism, postoperative pain, inflammatory markers

## Abstract

**Background:** Bariatric surgery induces a systemic inflammatory response to surgical stress that may influence postoperative pain and analgesic requirements, and CBC-derived inflammatory indices can reflect preoperative and postoperative immunometabolic dynamics related to this response. This retrospective observational search evaluated whether inflammatory indices derived from routine complete blood counts are associated with postoperative pain and opioid consumption after ultrasound-guided transversus abdominis plane (TAP) block in patients with morbid obesity. **Methods:** In this retrospective observational search, 120 patients undergoing bariatric surgery were divided into two groups: TAP block (Group T, n = 59) and local infiltration of the trocar insertion site (Group K, n = 61). Preoperative and postoperative 24-h hematological indices, VAS pain scores (0, 2, 6, 12, 24 h), and cumulative 24-h opioid consumption were analyzed. **Results:** Group T had significantly lower 24-h opioid consumption and VAS scores at all time points. Regarding changes between groups, the increase in neutrophil counts was greater in Group T than in Group K, whereas the change in SIRI values was smaller in Group T compared to Group K. ROC analysis showed no clinical benefit for NLR, PLR, SII, or SIRI. Only PCT (Plateletcrit) showed weak inverse discriminative ability. **Conclusions:** Compared with trocar site local anesthetic infiltration using an equivalent bupivacaine dose, TAP block was associated with lower postoperative pain and opioid consumption. The smaller postoperative rise in SIRI observed in the TAP block group is an exploratory, hypothesis-generating finding that did not survive correction for multiple comparisons and should not be interpreted as evidence of a causal anti-inflammatory effect. Overall, CBC-derived inflammatory indices did not perform as reliable predictive biomarkers for postoperative analgesic outcomes in this bariatric surgery population.

## 1. Introduction

Morbid obesity is increasingly recognized not merely as a condition of excessive body weight but as a chronic inflammatory and metabolic disorder. Expansion of adipose tissue is accompanied by adipocyte hypertrophy, immune-cell infiltration, and increased release of proinflammatory mediators, contributing to a persistent state of low-grade systemic inflammation [1,2,3]. This altered immunometabolic environment may influence the physiological response to surgical stress and contribute to interindividual variability in postoperative pain intensity and analgesic requirements.

Laparoscopic sleeve gastrectomy (LSG) is a surgical method used in the treatment of morbid obesity. TAP block is an important component of multimodal analgesic strategies in these patients. Studies have shown that TAP block can improve postoperative pain control; the magnitude of its analgesic effect appears to vary among patients [4,5,6]. This variability suggests that, in addition to technical and perioperative factors, patient-specific biological characteristics may influence postoperative analgesic response.

Hematological parameters derived from routine complete blood count may provide practical and inexpensive markers of the underlying inflammatory state. Plateletcrit (PCT) reflects the total circulating platelet mass. Beyond their well-established role in hemostasis, platelets are also involved in inflammatory signaling, endothelial activation, and immune regulation. Higher PCT and platelet-to-lymphocyte ratio (PLR) values have been reported in patients with morbid obesity compared with normal-weight individuals, and associations between obesity, platelet activation, and systemic inflammation have also been demonstrated [7,8].

The neutrophil-to-lymphocyte ratio (NLR) and PLR are readily calculated indices that reflect different components of systemic inflammatory and immune responses [9,10]. More recently, the systemic immune-inflammation index (SII) and systemic inflammation response index (SIRI) have been introduced as composite markers incorporating neutrophil, lymphocyte, platelet, and monocyte counts to provide a broader assessment of systemic inflammatory activity [11,12,13]. Because all these parameters can be derived from routine blood counts without additional laboratory testing, their assessment in both the preoperative and postoperative periods may offer a practical means of evaluating perioperative inflammatory changes. However, data remain limited regarding the perioperative changes in PCT, NLR, PLR, SII, and SIRI in patients with morbid obesity and their relationship with postoperative pain and analgesic requirements after TAP block.

The aim of this study was to evaluate the changes in PCT, NLR, PLR, SII, and SIRI levels before and after ultrasound-guided TAP block in patients undergoing bariatric surgery and to examine the relationships between these inflammatory indices and postoperative pain scores and analgesic requirements.

## 2. Materials and Methods

### 2.1. Study Design and Patient Population

**Figure 1:**
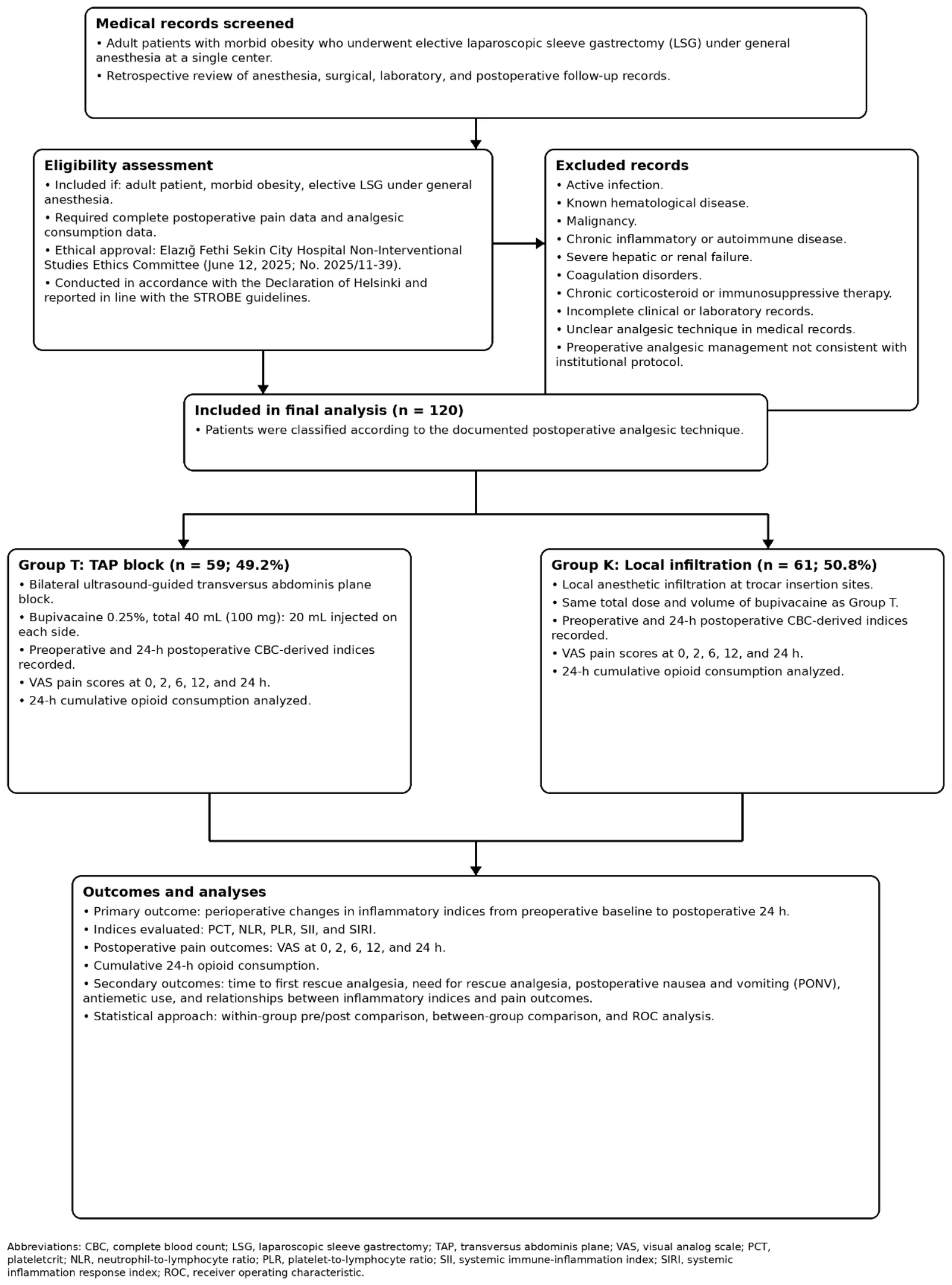
Study flowchart.

This single-center, retrospective observational study included adult patients with morbid obesity (body mass index [BMI] ≥ 40 kg/m^2^, or ≥35 kg/m^2^ with an obesity-related comorbidity) who underwent elective LSG under general anesthesia between 1 January 2021 and 31 May 2025, and who had complete postoperative pain and analgesic consumption data. Patients who experienced infections affecting inflammatory markers, those with hematological diseases, those using steroids, those with surgical complications, or whose records etc were not fully accessible were excluded from the search. Ethical approval was obtained from the Elazığ Fethi Sekin City Hospital Non-Interventional Studies Ethics Committee (date: 12 June 2025; approval no.: 2025/11-39). Patients were divided into two groups according to the postoperative analgesic technique documented in the anesthesia and surgical records (TAP block and trocar-site local infiltration). Prior to January 2023, infiltration anesthesia was typically administered to trocar insertion sites by the surgical team. From that date onwards, the administration of a TAP block for analgesia in suitable patients was adopted as the anesthesia department’s protocol. Due to the retrospective nature of our study, randomization was not performed. The study was conducted in accordance with the principles of the Declaration of Helsinki and reported in accordance with the Strengthening the Reporting of Observational Studies in Epidemiology (STROBE) guidelines. The study flowchart is presented in Figure 1.

Cases with active infection, known hematological disease, malignancy, chronic inflammatory or autoimmune disease, severe hepatic or renal failure, coagulation disorders, chronic corticosteroid or immunosuppressive therapy, or incomplete clinical or laboratory records were excluded from the study. Patients whose analgesic technique could not be clearly determined in their medical records or whose preoperative analgesic administration differed significantly from the institutional protocol were also excluded. Due to the retrospective design, no additional interventions or blood samples were taken for the study.

Patients who underwent bilateral TAP block constituted the TAP group (Group T), while patients who received local anesthetic infiltration at the trocar insertion sites formed the local infiltration group (Group K), thus forming two groups.

### 2.2. Anesthesia Procedure

All patients were evaluated by an anesthesiologist preoperatively according to the institutional anesthesia protocol. Patients underwent standard anesthesia monitoring following the appropriate fasting period. Laparoscopic sleeve gastrectomy was performed under general anesthesia. Surgery duration, anesthesia duration, intraoperative analgesic administration, and other relevant perioperative variables were retrospectively obtained from anesthesia and surgical records at the end of surgery.

In the TAP block group, following skin antisepsis and sterile preparation, a linear ultrasound transducer was placed in the lateral abdominal wall between the costal margin and the iliac crest. Under ultrasound guidance, the external oblique, internal oblique, and transversus abdominis muscles were identified. Using an in-plane approach, the needle tip was advanced into the fascial plane between the internal oblique and transversus abdominis muscles. Hydrodissection was used to confirm correct needle placement. A total of 40 mL of 0.25% bupivacaine (100 mg) was administered bilaterally under direct ultrasound guidance; 20 mL (50 mg) was injected into each side.

In the local infiltration group, local anesthetic infiltration was applied to the trocar insertion sites at the end of surgery as part of the routine postoperative analgesia protocol. The total dose and volume of bupivacaine were identical to those used in the TAP group. Thus, both groups received equivalent total doses and volumes of bupivacaine, differing only in the anatomical region and local anesthetic application technique. Following the completion of the analgesia procedure, neuromuscular blockade was reversed according to routine clinical practices.

Postoperative analgesia was administered according to a standardized multimodal analgesia protocol. Rescue analgesics were given when clinically necessary according to postoperative pain scores and routine clinical assessment. The type, timing, and total dosage of postoperative analgesics were obtained from medication and nursing records. Postoperative pain intensity was assessed using the visual analog scale (VAS) scores recorded as part of routine postoperative follow-ups. VAS scores obtained at predefined postoperative time points were retrospectively retrieved from patient files and the hospital’s electronic medical record system. Ondansetron and/or metoclopramide were administered instead of dexamethasone for postoperative nausea and vomiting (PONV), as this would affect the study data. In total, 1000 mg of paracetamol was used for rescue analgesia for 8 h. Tramadol was used as an opioid when necessary for rescue analgesia, and 24-h opioid consumption was calculated using the amount of tramadol used (mg). Patients receiving nonsteroidal anti-inflammatory drugs were excluded from the study due to the potential for them to affect the study results.

### 2.3. Laboratory Measurements and Inflammatory Indices

Preoperative inflammatory indices were calculated using routine blood samples taken before surgery, while postoperative indices were calculated using blood samples taken 24 h postoperatively. As our search was retrospective, no additional blood samples were collected. Platelet count, mean platelet volume, neutrophil count, lymphocyte count, monocyte count, and plateletcrit (PCT) were recorded from complete blood count results, whereas NLR, PLR, SII, and SIRI were calculated from the corresponding complete blood count parameters at the respective preoperative and 24-h postoperative times.

All indices were calculated using cell counts obtained from the same blood sample at each time point. Preoperative and 24-h postoperative PCT, NLR, PLR, SII, and SIRI values were compared both within each group and between TAP and local infiltration groups to assess perioperative changes in systemic inflammatory activity. Platelet count, mean platelet volume, neutrophil count, lymphocyte count, monocyte count, and plateletcrit (PCT) were recorded from complete blood count results, whereas NLR, PLR, SII, and SIRI were calculated from the corresponding complete blood count parameters at the respective preoperative and 24-h postoperative times using the following standard formulas: NLR = neutrophil count/lymphocyte count; PLR = platelet count/lymphocyte count; SII = (platelet count × neutrophil count)/lymphocyte count; SIRI = (neutrophil count × monocyte count)/lymphocyte count.

### 2.4. Study Outcomes

The primary objective was to compare perioperative changes in PCT, NLR, PLR, SII, and SIRI between patients who underwent ultrasound-guided TAP block and those who underwent trocar-site local anesthetic infiltration and to investigate whether these inflammatory indices were associated with postoperative analgesic outcomes.

#### 2.4.1. Primary Outcome

Changes in PCT, NLR, PLR, SII, and SIRI were observed from the preoperative period to 24 h postoperatively, and whether these changes differed between the TAP and local infiltration groups was noted.

Postoperative VAS scores.

Cumulative postoperative analgesic consumption.

#### 2.4.2. Secondary Outcomes 

The secondary outcomes included the following:

Time to first rescue analgesia;

Need for rescue analgesia;

The relationships between inflammatory indices and postoperative pain outcomes.

### 2.5. Statistical Analysis

Statistical analyses were performed using IBM SPSS Statistics software (version 25.0, IBM Corp., Armonk, NY, USA). A *p*-value < 0.05 was considered significant. The normality of continuous variables was assessed using the Kolmogorov–Smirnov test (with Lilliefors correction) and the Shapiro–Wilk test. Normally distributed continuous variables are presented as mean ± standard deviation (SD), and non-normally distributed continuous variables as median (interquartile range, IQR); categorical variables are presented as numbers (n) and percentages.

For comparison of repeated measurements obtained before (preoperative) and after (postoperative) surgery in the same patients, within each group separately, according to the normality of both the preoperative and postoperative values as assessed by the Shapiro–Wilk test, either the paired-samples t test (if both measurements were normally distributed) or the Wilcoxon signed-rank test (if at least one measurement was not normally distributed) was utilized. The change in VAS pain scores across five time points (0, 2, 6, 12, and 24 h) was first evaluated holistically, within each group separately, using the Friedman test; when the overall model was significant, each time point was compared pairwise with the baseline (0 h) value using the Wilcoxon signed-rank test (the Wilcoxon test was preferred for all time-point comparisons because the VAS is an ordinal scale).

For between-group comparisons, either the independent-samples t test (for normally distributed variables) or the Mann–Whitney U test (for non-normally distributed variables) was used for variables evaluated separately in each group. Homogeneity of variances was assessed using the Levene test; when variances were not homogeneous, the t-value calculated with corrected degrees of freedom was reported.

The ability to predict “high/low opioid consumption” and “high/low VAS at 6 h” (binary data set to median) was assessed using receiver operating characteristic (ROC) curve analysis (area under the curve [AUC], 95% confidence interval); as a complementary, non-dichotomized approach, Spearman correlation coefficients between each preoperative inflammatory index and the continuous values of 24-h opioid consumption and VAS at 6 h were also calculated. Because approximately 40 significance tests were performed across the hematological, VAS, and ROC comparisons without correction for multiple testing, findings are additionally interpreted against a Bonferroni-corrected threshold (α = 0.05/13 = 0.0038 for the 13 hematological markers) and, where relevant, are described as exploratory/hypothesis-generating rather than confirmatory. To evaluate whether the between-group difference in the magnitude of change (Δ) for neutrophil count and SIRI was robust to baseline imbalance and other potential confounders, multivariable linear regression models were additionally fitted for each Δ marker, adjusting for the corresponding preoperative marker level, age, sex, and surgical duration. Since significant differences existed between Group T and Group K regarding ASA physical status class, body weight, and BMI, these three variables were also compared between the groups: Fisher’s exact test was used for ASA class, while the Mann–Whitney U test (based on the Shapiro–Wilk normality test) was used for body weight and BMI. To assess whether this additional baseline imbalance influenced the findings regarding inflammatory markers or analgesic outcomes, the Δneutrophil and ΔSIRI regression models were reconstructed by including ASA class and BMI as covariates.

## 3. Results

A total of 120 patients were included in the study (Group T: n = 59; Group K: n = 61). No significant difference was found between the two groups with respect to age (*p* = 0.962), sex distribution (*p* = 0.699), height (*p* = 0.858), diabetes mellitus (*p* = 0.150), hypertension (*p* = 0.166), OSAS (*p* = 1.000), and smoking status (*p* = 0.439) (Table 1). However, body weight (Mann–Whitney U = 1327.5, *p* = 0.013) and BMI (Mann–Whitney U = 1266.5, *p* = 0.005) were significantly higher in Group K, and ASA physical status class was significantly imbalanced between groups (Fisher’s exact test, *p* = 0.006): Group T was predominantly ASA II (62.7%), whereas Group K was predominantly ASA III (63.9%). Because ASA class reflects overall comorbidity burden and could plausibly confound both the inflammatory-marker and analgesic-outcome comparisons, we performed additional sensitivity analyses adjusting for ASA class and BMI (Table 1).

A bilateral TAP block was applied to 49.2% (n = 59) of patients, while the remaining 50.8% (n = 61) constituted the local infiltration group. Surgical duration (*p* = 0.301), anesthesia duration (*p* = 0.856), and the distribution of postoperative complications (*p* = 0.631) did not differ significantly between groups; no complication was observed in 90.0% (n = 108) of patients, with atelectasis being the most common complication (5.0%, n = 6). In contrast, 24-h postoperative opioid consumption recorded was significantly lower in Group T than in Group K (median 100 mg [100–150] vs. 200 mg [150–200]; U = 350.0; *p* < 0.001), directly supporting the opioid-sparing effect of the TAP block. Details are presented in Table 2.

Hematologic and inflammatory markers measured before and after surgery were compared against their own baseline (preoperative) value separately within the group that received a TAP block (Group T, n = 59) and the local infiltration group (Group K, n = 61); the two groups were additionally compared with each other in terms of the magnitude of change (Δ, the postoperative–preoperative difference score).

Comparing the change score rather than the raw postoperative values was preferred because the two groups already differed significantly at the preoperative level; comparing the change score prevents this baseline imbalance from confounding the postoperative comparison and more accurately reflects the true effect of the TAP block on the magnitude of the inflammatory response. In both groups, a statistically significant postoperative change from baseline was found for the great majority of the markers examined (*p* < 0.05). Two exceptions to this general pattern are noteworthy: MPV increased significantly from baseline only in Group K (Wilcoxon, *p* = 0.002 *), with no significant change in Group T (paired-samples t test, *p* = 0.272); PDW decreased significantly only in Group K (Wilcoxon, *p* < 0.001), with no significant change in Group T (Wilcoxon, *p* = 0.187). This subgroup-level discrepancy shows that a single “change-from-baseline” *p*-value calculated across the total population may in fact represent the average of two genuinely different subgroup patterns and may not be sufficiently informative on its own. Preoperatively, lymphocyte, CRP, WBC, PLT, and PCT values were higher in Group T, whereas SIRI and NLR were significantly lower (detailed *p*-values are presented in Table 1 and Table 3); because this baseline imbalance could bias a direct comparison of raw postoperative values, the between-group comparison was performed using the change (Δ) score. In this comparison, of the 13 markers evaluated, the magnitude of change differed significantly between the groups for only two markers: neutrophil count (Mann–Whitney, *p* = 0.037 *) and SIRI (Mann–Whitney, *p* = 0.047 *); for the remaining 11 markers (lymphocyte, monocyte, CRP, MPV, WBC, PLT, PDW, PCT, NLR, PLR, and SII), no statistically significant difference was found between the groups in the magnitude of change (Table 3). This finding is important: some differences that appeared significant in the earlier comparison based on raw postoperative values (notably lymphocyte, PLT, and PCT) may, in fact, reflect the baseline (preoperative) imbalance between the groups; the true magnitude of the inflammatory response for these markers is similar between the two groups. In contrast, although neutrophil counts were also high in Group T, the magnitude of the increase in SIRI was significantly greater in Group K than in Group T; this may indicate that TAP blockade somewhat reduced the systemic inflammatory response (Table 3). In the postoperative period compared to the preoperative period, all hematological measurements—except for MPV and PDW (where no difference was observed)—were found to be higher in both groups; however, between the groups, SIRI was lower (Mann–Whitney, *p* = 0.047) and neutrophil count was higher (Mann–Whitney, *p* = 0.037). Detailed *p*-values are presented in Table 1 and Table 3. For the remaining 11 markers (lymphocyte, monocyte, CRP, MPV, WBC, PLT, PDW, PCT, NLR, PLR, and SII), no statistically significant difference was found between the groups in the magnitude of change (Table 3). Because this baseline imbalance could bias a direct comparison of raw postoperative values, the between-group comparison was performed using the change (Δ) score. This finding is important: some differences that appeared significant in the earlier comparison based on raw postoperative values (notably lymphocyte, PLT, and PCT) may, in fact, reflect the baseline (preoperative) imbalance between the groups; the true magnitude of the inflammatory response for these markers is similar between the two groups. The apparently discordant pattern for neutrophil count and SIRI is explained by the fact that SIRI is a nonlinear composite of neutrophil, monocyte, and lymphocyte counts rather than a simple reflection of neutrophil count alone: the postoperative rise in monocyte count was smaller in Group T than in Group K (median Δ 0.20 vs. 0.23 × 10^9^/L, *p* = 0.175) and the fall in lymphocyte count was similar between groups (median Δ −0.85 vs. −0.77 × 10^9^/L, *p* = 0.631), so a larger neutrophil rise in Group T did not translate into a larger SIRI rise. Because approximately 40 statistical tests were performed across this study without correction for multiple comparisons, neither the neutrophil nor the SIRI between-group difference would remain significant after Bonferroni correction (α = 0.0038 for 13 markers), and both findings should be regarded as exploratory rather than confirmatory. To assess whether these two differences were robust to the baseline im-balance between groups, multivariable linear regression models for Δneutrophil and ΔSIRI were fitted, adjusting for the corresponding preoperative marker level, age, sex, and surgical duration; the between-group difference remained statistically significant for both Δneutrophil (adjusted β = 1.28 × 10^9^/L favoring a larger rise in Group T, *p* = 0.012) and ΔSIRI (adjusted β = −1.36 favoring a smaller rise in Group T, *p* = 0.029), suggesting that these differences are not fully explained by the measured baseline imbalance, age, sex, or surgical duration, although unmeasured confounders (e.g., corticosteroid use) cannot be excluded. This may indicate that TAP blockade is associated with a somewhat smaller systemic inflammatory response as reflected by SIRI, but given the lack of correction for multiple comparisons, this should be interpreted as hypothesis-generating (Table 3).

Individual informed consent was not obtained, as this retrospective study was based on existing medical records.

Postoperative pain level was assessed by VAS at 0, 2, 6, 12, and 24 h. In both groups, the overall change across the five time points was significant by the Friedman test (Group T: χ^2^ = 169.60, *p* < 0.001; Group K: χ^2^ = 222.49, *p* < 0.001). In pairwise comparisons with the 0 h value, Group T showed a significant decline in VAS at every time point (even at 12 h, albeit with borderline significance; *p* = 0.010); in Group K, by contrast, the 12 h VAS value was not significantly different from the 0 h value (*p* = 0.202), indicating that pain intensity in this group had not yet declined significantly from its initial postoperative level by 12 h. Furthermore, VAS scores were statistically significantly lower in Group T at every time point evaluated (Table 4); the most pronounced difference was observed in the early postoperative period (2 and 6 h). These findings suggest that the TAP block provides a marked analgesic effect, particularly in the early postoperative period, and that pain intensity declined both more and sooner in Group T than in Group K.

**Figure 2:**
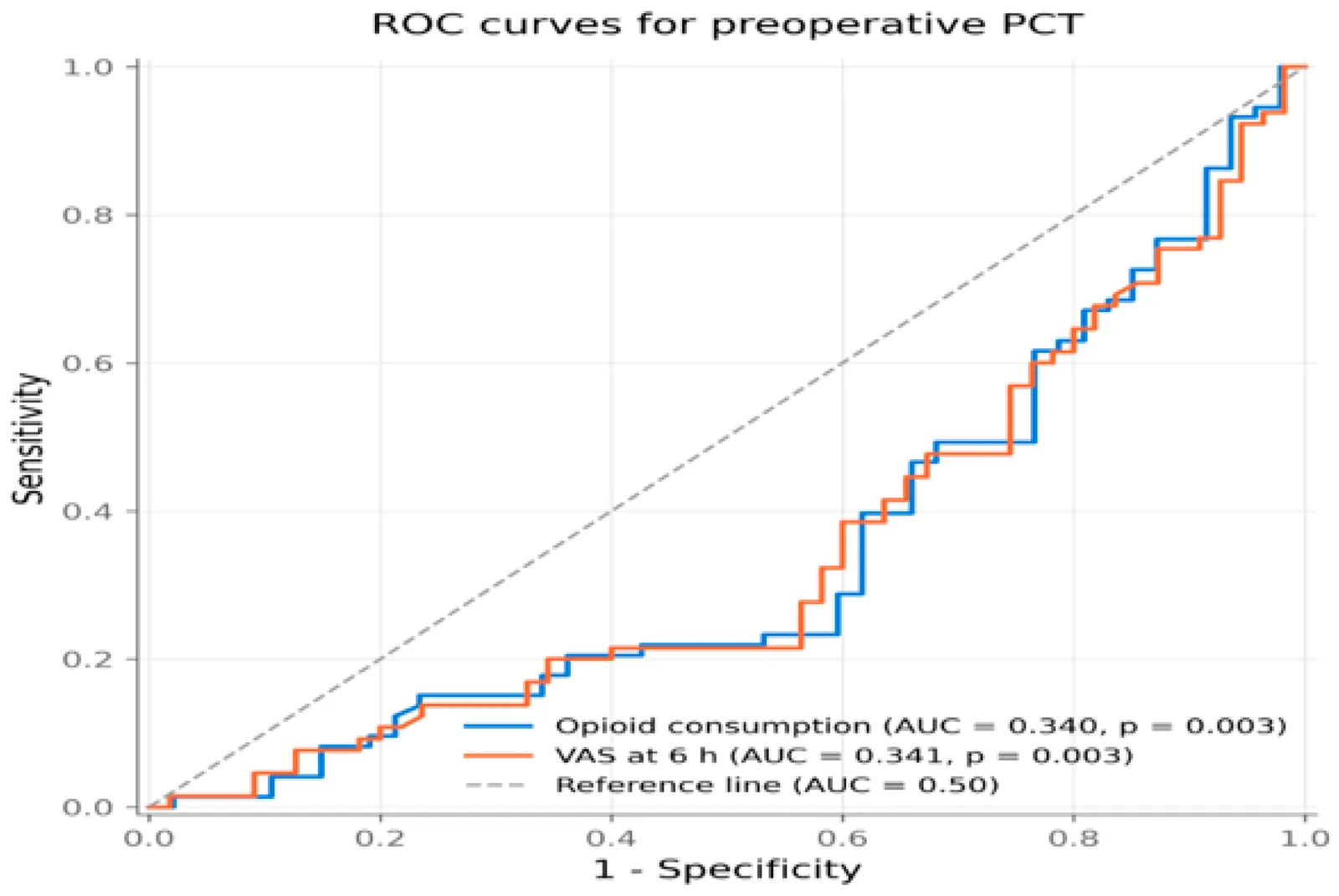
ROC curves of preoperative PCT for predicting high opioid consumption and a high VAS pain score at 6 h.

The discriminative ability for predicting “high/low opioid consumption” and “high/low VAS at 6 h” (dichotomized at the median) was assessed by ROC analysis (Table 5). Of the five indices, only PCT showed a statistically significant area under the curve (AUC); however, this area fell below 0.50 (AUC = 0.340, *p* = 0.003 for opioid consumption; AUC = 0.341, *p* = 0.003 for VAS at 6 h), meaning that PCT shows discriminative ability in the reverse direction (lower PCT values indicate a higher probability of greater pain/opioid consumption; when the direction is reversed, the effective AUC is approximately 0.66) (Figure 2). None of the other four indices (NLR, PLR, SII, SIRI) showed statistically significant discriminative ability (all *p* > 0.12). Overall, none of the indices examined reached the AUC ≥ 0.70 threshold conventionally considered clinically useful. As a complementary, non-dichotomized analysis, Spearman correlations between each preoperative index and the continuous outcomes were consistent with these ROC findings: PCT correlated inversely with both opioid consumption (ρ = −0.27, *p* = 0.003) and VAS at 6 h (ρ = −0.23, *p* = 0.011); NLR, PLR, and SII showed no significant correlation with either outcome (*p* ≥ 0.15); and SIRI showed a weak, nominally significant correlation with VAS at 6 h (ρ = 0.20, *p* = 0.029) but not with opioid consumption (*p* = 0.080). None of these correlations would remain significant after Bonferroni correction for the 10 tests performed (α = 0.005). The ROC curves obtained for PCT are shown in Figure 2; both curves lie below the reference diagonal, visually confirming this reverse-direction but statistically significant discriminative ability.

Because Group T and Group K also differed significantly in ASA physical status class, body weight, and BMI, we performed a further sensitivity analysis adding ASA class and BMI as covariates to the Δneutrophil and ΔSIRI regression models. After this additional adjustment, the between-group effect on Δneutrophil weakened to borderline significance (adjusted β = 1.08 × 10^9^/L, *p* = 0.050), while the effect on ΔSIRI remained significant (adjusted β = −1.37, *p* = 0.045); ASA class and BMI were not independently significant predictors in either model (all *p* > 0.22). Full model output for all six models is presented in Table 6 below.

## 4. Discussion

The analysis revealed no significant between-group differences in demographic characteristics, postoperative complications, or surgical duration. Moreover, 24-h opioid consumption decreased significantly after TAP block, and pain scores (VAS) were lower at all measurement times, most notably at 2 and 6 h. Because of baseline differences between groups, between-group comparisons were based on the change (Δ) score, which showed a significantly larger increase in neutrophil count (*p* = 0.037) but a significantly smaller increase in SIRI (*p* = 0.047) in Group T than in Group K; neither difference survived correction for multiple comparisons, although both persisted after multivariable adjustment (see Section 3). In ROC and correlation analysis, none of the indices reached the clinical validity threshold (AUC ≥ 0.70); only PCT showed weak inverse predictive power (AUC = 0.340; *p* = 0.003).

Obesity is a growing problem today. Many factors contribute to obesity, including high-calorie foods, sedentary lifestyles, genetic factors, and eating disorders. According to the Ministry of Health, Türkiye’s obesity rate is 17% [14]. Obesity has the potential to cause cardiovascular diseases (hypertension, heart failure, arrhythmias, etc.), osteoarthritis, kidney problems, and metabolic syndrome. Therefore, while non-surgical methods such as diet and exercise are treatment options, surgery is also an option when necessary.

Effective postoperative analgesia is an essential component of perioperative care. For this purpose, in addition to systemic analgesics, local anesthetics are administered to the trocar insertion sites, and the TAP block has been used since the early 2000s. In our comparison of TAP block with trocar-site local anesthetic infiltration, we observed that opioid consumption decreased by approximately half in patients who underwent TAP block, and the VAS scores decreased significantly at all measurement times. McCarthy et al. also obtained similar results in their bariatric studies. Studies by Arik et al. and Chaw et al. showing that the TAP block reduces analgesic and opioid consumption are also available in the literature [15,16]. Opioids, particularly morphine, may also modulate inflammatory and immune responses. They suppress neutrophil function by inhibiting pro-inflammatory secretions such as TNF-alpha (Tumor Necrosis Factor Alpha), Interleukin-1 beta (IL-1β), Interleukin-2 (IL-2), Interleukin-6 (IL-6), Interleukin-8, and by increasing cortisol synthesis via the hypothalamus–pituitary–adrenal axis, thereby suppressing the immune system [17,18]. While these effects are generally long-lasting, it should be remembered that short-term use, such as post-surgical use, can also suppress the immune system. From this perspective, by reducing opioid consumption, the TAP block may help limit opioid-related immunosuppression and could thereby have a favorable, though as-yet-unproven, effect on postoperative recovery. Conversely, because opioids can suppress immune function, the higher opioid consumption seen in the local infiltration group could theoretically be associated with a greater risk of infection; this hypothesis was not directly tested in the present study and requires prospective confirmation.

In the analysis of our study data, VAS values were lower in patients undergoing TAP block at all measurement times, although the difference was more pronounced at 2 and 6 h. Alsharari et al. also stated in their study that TAP block provided similarly effective analgesia [19]. In their study comparing TAP block with Erector Spinae Plane (ESP) block in major abdominal surgeries, Karatepe et al. also stated that while not as effective as ESP, TAP block provided sufficient analgesia and reduced opioid consumption [20]. These findings may suggest that the reduction in pain, along with increased patient satisfaction, reduced risk of breathing difficulties and atelectasis due to pain, reduced surgical and psychological stress, and faster recovery due to the decrease in both opioid side effects and immune system suppression.

Essentially, obesity is characterized by chronic low-grade systemic inflammation. Due to increased fat tissue, tissue-level hypoxia leads to lipolysis, increased macrophage activity, and increased levels of cytokines such as TNF-alpha, IL-1β, and IL-6, all of which contribute to low-level inflammation. Furthermore, it increases insulin resistance, predisposing patients to diabetes, while elevated leptin (which increases platelet aggregation) and PAI-1 (Plasminogen Activator Inhibitor-1) increase the risk of thrombosis. Surgical stress causes an increase in cortisol and catecholamines, leading to neutrophilia and increased catecholamine levels. Blocking the pain pathway reduces this increase, but post-operative changes in inflammatory responses such as neutrophilia and lymphopenia persist. In our study, although the significant changes shown in Table 3 occurred in both groups, neutrophil count was higher, and SIRI was lower in the TAP block group.

The effectiveness of TAP block on neutrophil counts varies according to studies. While there was a general increase in neutrophil counts in the postoperative period, as in our study, Espadas-González et al. reported no difference in neutrophil counts between the TAP-treated and non-treated groups, and Wang et al. reported lower neutrophil counts in the TAP block group [21,22]. In our study, neutrophil counts were higher in the TAP block group compared to the infiltration group. Unlike the studies by Wang and Espadas-González, our study involved the application of infiltration anesthesia. While neutrophil counts normally decrease due to reduced cortisol levels and other reasons related to the blockade of pain pathways, in our study, the hydrodissection of the TAP block and the entry of non-circulating storage neutrophils into circulation may have contributed to the effect [23]. Although the postoperative rise in neutrophil count was larger in the TAP block group in our study, this increase did not translate into a larger rise in the composite SIRI—which was, in fact, smaller in Group T—so it should not, on its own, be interpreted as evidence of greater overall inflammation in the TAP block group. Taken together with the monocyte and lymphocyte data described above, the neutrophil finding more plausibly reflects a technique-specific mechanism (e.g., hydrodissection-related mobilization of marginated neutrophils) than a net increase in systemic inflammatory burden.

In our study, the elevated lymphocyte count, platelet count, SIRI, NLR, PLR, PCT, and white blood cell count in both groups were considered a normal change due to surgical stress, independent of anesthesia. However, there was no difference between the groups. Studies on this subject are quite limited. In the study by Koksal et al., which compared general anesthesia with peripheral nerve block, a significant decrease in NLR, WBC (white blood cell count), and PLR was found [24]. In this study, no intra-group postoperative or pre-operative evaluation was performed. In a similar study by Yukselen et al., although NLR, PLR, and MPV values were significantly higher in the block group postoperatively, the groups were again similar in nature [25]. In their study evaluating NLR and PLR, Shukla et al. similarly found a lower rate in the block group among patients who underwent a brachial plexus block under general anesthesia [26]. In their studies, Yaslı et al. stated that PCT was significantly associated with postoperative pain [27]. In our study, the increases in the postoperative period were due to surgical stress playing a more active role in the increase, and neither the TAP block nor infiltration anesthesia affected visceral pain. Another factor is that, since studies are generally compared with general anesthesia, the analgesic effect of TAP block creates a potential for higher values of sterile inflammatory markers secondary to pain. In addition, the effect of the chronic inflammation process present at baseline levels in obese individuals on these parameters should be considered. In our study, although SIRI levels were higher in both groups during the postoperative period, they were lower in the TAP block group than in the local infiltration group. We believe this is due to the fact that the analgesic efficacy of the TAP block is greater and covers a wider area, thus suppressing the relatively higher levels of pain-related cortisol and cytokines, and consequently, the inflammation is less severe.

In the ROC analysis performed, although plateletcrit appeared to be significantly inversely related to pain, this relationship can be considered weak; therefore, the level of evidence is low. No significant relationship was found in terms of NLR, PLR, SII, and SIRI.

### Limitations

This study has several limitations. First, its single-center, retrospective observational design limits causal inference and may be associated with selection and information bias. Although between-group comparisons were based on change (Δ) scores to account for baseline differences in several inflammatory parameters, the possibility of residual confounding cannot be entirely excluded. Second, the inflammatory assessment was limited to routinely available complete blood count-derived indices measured preoperatively and at 24 h postoperatively. Therefore, earlier postoperative inflammatory changes and more specific inflammatory mediators, such as cytokines, could not be evaluated. Third, postoperative pain scores and analgesic consumption were obtained retrospectively from routine clinical records, which may be less precise than prospectively standardized measurements. Fourth, because TAP block was compared with trocar-site local anesthetic infiltration using the same total dose and volume of bupivacaine, the findings reflect the relative effects of these two analgesic approaches rather than the effect of TAP block compared with placebo or no regional analgesic intervention. Fifthly, the modest sample size and single-center setting may also limit the generalizability of the findings. Regarding obesity classification, patients with a BMI > 40, as well as those with a BMI between 30 and 40 whose diabetes mellitus (DM) and hypertension (HT) are under control, are included in this scope. Since bariatric surgery is an elective procedure, the operation is scheduled only after comorbidities such as DM and HT have been brought under control. Our study was retrospective in nature, and only patients with complete outcome data were included; this can be considered a limitation of the study. When analyzed in terms of BMI values, the patient groups were clinically similar (Group T: 43.23; Group K: 46.87; Total: 44.6). Finally, although BMI has the potential to constitute a study limitation, we believe it is highly unlikely to have had a significant impact on inflammation markers.

## 5. Conclusions

Our search showed that TAP block was associated with significantly lower pain compared with infiltration anesthesia performed at the trocar entry sites (at an equivalent dose) in patients undergoing laparoscopic obesity surgery. Although postoperative elevations in markers such as NLR, PLR, SII, and SIRI were observed in both groups, the smaller increase in SIRI in the TAP block group is an exploratory finding—not surviving correction for multiple comparisons—that suggests, but does not establish, a partial anti-inflammatory effect and warrants confirmation in prospective studies. Furthermore, none of the other CBC-derived indices examined performed adequately as predictors of clinical outcomes, pain, or opioid requirement in this population.

## Figures and Tables

**Table 1:** Comparison of patients’ demographic and clinical characteristics by TAP group.

Variable	TAP-Yes (n = 59)	TAP-No (n = 61)	Total (n = 120)	Test	*p*
Age (years)	32 (25–44)	33 (25–39)	32 (25–40.8)	U = 1808.5	0.964
Sex, n (%)					
Male	18 (30.5%)	21 (34.4%)	39 (32.5%)		
Female	41 (69.5%)	40 (65.6%)	81 (67.5%)	Fisher	0.699
Body weight (kg)	118.0 (108.0–135.0)	125.0 (121.0–135.0)	123 (114.3–135)	U = 1327.5	0.013 *
Height (cm), mean ± SD	166.1 ± 9.0	165.8 ± 8.3	166 ± 8.6	t = 0.179	0.858
Body mass index (kg/m^2^)	43.23 (41.24–46.87)	46.11 (43.08–49.25)	44.6 (42.1–47.9)	U = 1266.5	0.005 *
ASA class, n (%)					
ASA II	37 (62.7%)	22 (36.1%)	59 (49.2%)		
ASA III	22 (37.3%)	39 (63.9%)	61 (50.8%)	Fisher	0.006 *
Diabetes mellitus, n (%)					
Yes	12 (20.3%)	20 (32.8%)	32 (26.7%)		
No	47 (79.7%)	41 (67.2%)	88 (73.3%)	Fisher	0.150
Hypertension, n (%)					
Yes	14 (23.7%)	22 (36.1%)	36 (30.0%)		
No	45 (76.3%)	39 (63.9%)	84 (70.0%)	Fisher	0.166
OSAS, n (%)					
Yes	5 (8.5%)	5 (8.2%)	10 (8.3%)		
No	54 (91.5%)	56 (91.8%)	110 (91.7%)	Fisher	1.000
Smoking, n (%)					
Yes	42 (71.2%)	39 (63.9%)	81 (67.5%)		
No	17 (28.8%)	22 (36.1%)	39 (32.5%)	Fisher	0.439

Continuous variables are presented as median (interquartile range) or mean ± standard deviation, as appropriate based on the Shapiro–Wilk test; categorical variables are presented as n (%). U: Mann–Whitney U test; t: independent-samples t test; Fisher: Fisher’s exact test (used for all categorical comparisons due to small expected cell counts in some strata). ASA: American Society of Anesthesiologists physical status classification; OSAS: obstructive sleep apnea syndrome. * *p* < 0.05 was considered statistically significant.

**Table 2:** Comparison of surgical/anesthesia durations, postoperative opioid consumption, and complications by TAP group.

Variable	TAP-Yes (n = 59)	TAP-No (n = 61)	Total (n = 120)	Test	*p*
Surgical duration (min)	65.0 (62.0–71.0)	65.0 (62.0–68.0)	65 (62–68.8)	U = 1603	0.301
Anesthesia duration (min)	75.0 (72.0–81.0)	76.0 (73.5–78.0)	76 (72–79)	U = 1765	0.856
Postop. 24 h opioid consumption mg	100 (100–150)	200 (150–200)	150 (100–200)	U = 350.0	***<0.001***
Postoperative complication, n (%)				χ^2^ = 3.449	0.631
Atelectasis	3 (5.1%)	3 (4.9%)	6 (5.0%)		
Fever	1 (1.7%)	2 (3.3%)	3 (2.5%)		
Chest pain	1 (1.7%)	0 (0.0%)	1 (0.8%)		
Urinary tract infection	1 (1.7%)	0 (0.0%)	1 (0.8%)		
Wound infection	1 (1.7%)	0 (0.0%)	1 (0.8%)		
No complication	52 (88.1%)	56 (91.8%)	108 (90.0%)		

Continuous variables are presented as median (interquartile range) (U: Mann–Whitney U; χ^2^: Pearson chi-square). For postoperative complications, the Pearson chi-square test was used in a 6-category table in which 83.3% of cells had an expected count <5; this result should therefore be interpreted with caution. The unit of opioid consumption was not specified in the original dataset and should be confirmed by the author (possibly mg morphine equivalent).

**Table 3:** Change from baseline in preoperative–postoperative laboratory markers within each TAP group and between-group comparison of the magnitude of change (Δ).

Variable	TAP-Yes (n = 59)				TAP-No (n = 61)				*p* (Δ, Between Groups)
	Preop	Postop	*p* (Within)	Δ	Preop	Postop	*p* (Within)	Δ	
Neutrophil (10^9^/L)	5.4 (4.7–6.4)	10.4 (8.9–12.2)	<0.001 *	4.7 ± 3.4	5.1 (4.5–6.3)	8.8 (7.6–11.5)	<0.001 *	3.8 ± 2.5	0.037 *
Lymphocyte (10^9^/L)	2.7 (2.3–3.4)	1.8 (1.5–2.4)	<0.001 *	−0.85 (−1.27–0.47)	2.1 (1.9–2.6)	1.2 (1.1–1.7)	<0.001 *	−0.77 (−1.02–−0.60)	0.629
Monocyte (10^9^/L)	0.60 (0.49–0.70)	0.74 (0.60–0.96)	<0.001 *	0.20 (0.04–0.32)	0.66 ± 0.19	0.90 ± 0.28	<0.001 *	0.24 ± 0.27	0.176
CRP (mg/L)	3.4 (2.9–4.3)	13.7 (8.8–19.7)	<0.001 *	10.0 (5.6–14.6)	3.2 (2.7–3.6)	13.6 (12.4–15.4)	<0.001 *	10.1 (9.3–12.4)	0.307
MPV (fL)	9.91 ± 0.86	9.98 ± 0.88	0.272	0.07 ± 0.49	9.90 (9.50–10.30)	10.10 (9.60–10.40)	0.002 *	0.20 (−0.10–0.30)	0.199
SIRI	1.23 (0.90–1.57)	4.16 (2.82–6.09)	<0.001 *	3.11 (1.47–4.58)	1.63 (0.97–2.18)	5.99 (3.45–7.96)	<0.001 *	3.84 (2.45–6.35)	0.047 *
WBC (10^9^/L)	9.3 (8.1–10.9)	13.6 (11.6–14.9)	<0.001 *	4.5 (2.3–6.2)	8.5 (7.4–10.0)	12.1 (11.1–15.0)	<0.001 *	3.9 ± 2.4	0.548
PLT (10^9^/L)	340.0 ± 71.6	303.9 ± 74.5	<0.001 *	−33.0 (−58.5–−15.0)	275.0 (247.0–313.0)	234.0 (215.0–264.0)	<0.001 *	−37.4 ± 34.5	0.614
PDW	10.9 (10.0–12.5)	10.8 (9.6–12.2)	0.187	−0.13 ± 1.00	11.3 (10.7–12.3)	11.1 (10.2–11.9)	<0.001 *	−0.40 ± 0.65	0.094
PCT (%)	0.33 ± 0.07	0.30 ± 0.07	<0.001 *	−0.03 (−0.06–−0.01)	0.28 ± 0.06	0.24 ± 0.05	<0.001 *	−0.03 ± 0.03	0.936
NLR	2.03 (1.57–2.45)	5.31 (3.81–8.04)	<0.001 *	3.21 (1.54–5.55)	2.50 (1.76–2.98)	6.75 (4.57–8.73)	<0.001 *	4.08 (2.45–6.11)	0.199
PLR	124.1 (96.7–150.8)	158.7 (117.7–228.5)	<0.001 *	33.1 (15.3–75.4)	128.6 (97.8–148.5)	176.9 (125.2–202.7)	<0.001 *	47.6 (22.2–68.1)	0.315
SII	704.8 (515.9–871.2)	1611.2 (1042.2–2488.2)	<0.001 *	1018.1 (365.4–1828.0)	687.9 (469.7–802.9)	1624.0 (1096.0–2032.1)	<0.001 *	814.0 (460.1–1358.7)	0.527

Continuous variables are presented as mean ± standard deviation (if normally distributed) or median (interquartile range) (if not normally distributed), based on the Shapiro–Wilk test applied separately to the preoperative, postoperative, and Δ (postoperative–preoperative) values within each TAP subgroup. “*p* (within)”: paired preoperative–postoperative comparison within the given TAP subgroup. “Δ”: descriptive statistic of the per-patient change score (postoperative–preoperative) within the given TAP subgroup. “*p* (Δ, between groups)”: comparison of the Δ score between the TAP-Yes and TAP-No groups using the Mann–Whitney U test (chosen instead of comparing raw postoperative values, to exclude baseline imbalance). WBC: white blood cell count; PLT: platelet count; MPV: mean platelet volume; PDW: platelet distribution width; PCT: plateletcrit; NLR: neutrophil-to-lymphocyte ratio; PLR: platelet-to-lymphocyte ratio; SIRI: systemic inflammation response index; SII: systemic immune-inflammation index; CRP: C-reactive protein. * *p* < 0.05.

**Table 4:** Change from baseline in postoperative VAS pain scores within each TAP group and between-group comparison.

Time Point	TAP Group (n = 59)		Infiltration Group (n = 61)		*p* (Between Groups)
	VAS	*p* (Within)	VAS	*p* (Within)	
0 h (baseline)	2 (1–2)	Baseline	2 (2–3)	Baseline	<0.001 *
2 h	3 (2–3)	<0.001 *	4 (3–5)	<0.001 *	<0.001 *
6 h	3 (2–3)	<0.001 *	5 (4–6)	<0.001 *	<0.001 *
12 h	2 (1–2)	0.010 *	2 (2–3)	0.202	0.008 *
24 h	1 (0–1)	<0.001 *	1 (1–1)	<0.001 *	0.007 *
Friedman test (within- time overall model)	χ^2^ = 169.60; *p* < 0.001 *		χ^2^ = 222.49; *p* < 0.001 *		

* *p* < 0.05.

**Table 5:** Area under the ROC curve (AUC) of preoperative indices for predicting high opioid consumption and high VAS pain score at 6 h.

Index	AUC (Opioid)	95% CI (Opioid)	*p* (Opioid)	AUC (VAS-6 h)	95% CI (VAS-6 h)	*p* (VAS-6 h)
**PCT**	0.340	0.239–0.442	0.003 *	0.341	0.242–0.439	0.003 *
**NLR**	0.514	0.405–0.622	0.803	0.537	0.433–0.642	0.482
**PLR**	0.520	0.412–0.627	0.717	0.513	0.409–0.618	0.802
**SII**	0.447	0.340–0.554	0.328	0.462	0.358–0.566	0.474
**SIRI**	0.540	0.436–0.643	0.463	0.582	0.479–0.685	0.123

AUC: area under the curve; CI: confidence interval; H0: true area = 0.50. Opioid group: n = 120 (73 high, 47 low); VAS-6 h group: n = 120 (65 high, 55 low), dichotomized at the median. * *p* < 0.05.

**Table 6:** Multivariable regression analysis.

Predictor	B	SE	95% CI	β	*p*
Model 1: ΔNeutrophil (×10^9^/L)—R^2^ = 0.247, Adjusted R^2^ = 0.200, F(7112) = 5.248, *p* < 0.001					
Constant	13.202	4.205	4.871 to 21.533	—	0.002 *
TAP group (ref: infiltration)	1.080	0.545	0.000 to 2.161	0.180	0.050
Preoperative neutrophil count	−0.772	0.142	−1.054 to −0.491	−0.462	<0.001 *
Age (years)	−0.013	0.031	−0.075 to 0.050	−0.042	0.685
Sex	0.137	0.553	−0.958 to 1.232	0.021	0.804
Surgical duration (min)	−0.069	0.055	−0.179 to 0.040	−0.116	0.211
ASA class (ref: II)	−0.747	0.649	−2.032 to 0.539	−0.125	0.252
BMI (kg/m^2^)	0.004	0.060	−0.115 to 0.122	0.005	0.953
**Model 2: ΔSIRI—R^2^ = 0.213, Adjusted R^2^ = 0.163, F(7112) = 4.319, *p* < 0.001**					
Constant	5.108	4.946	−4.692 to 14.909	—	0.304
TAP group (ref: infiltration)	−1.365	0.674	−2.700 to −0.029	−0.189	0.045 *
Preoperative SIRI	−0.717	0.183	−1.079 to −0.355	−0.354	<0.001 *
Age (years)	−0.041	0.041	−0.122 to 0.041	−0.110	0.325
Sex	0.255	0.679	−1.091 to 1.601	0.033	0.709
Surgical duration (min)	−0.028	0.068	−0.162 to 0.105	−0.039	0.675
ASA class (ref: II)	−0.737	0.828	−2.377 to 0.904	−0.102	0.375
BMI (kg/m^2^)	0.091	0.074	−0.055 to 0.237	0.111	0.220

B: unstandardized regression coefficient; SE: standard error; CI: confidence interval; β: standardized coefficient. TAP group coded 1 = TAP block, 0 = local infiltration (reference category); positive B values indicate a larger increase in Group T relative to Group K. Models 1 and 2 correspond to the primary analysis described above; Models 1b and 2b add ASA class and BMI as covariates to assess robustness to the baseline imbalance in these variables (Table 1); and Models 3 and 4 apply the same ASA/BMI-adjusted approach to the two primary analgesic outcomes, 24 h opioid consumption and VAS at 6 h, as a further sensitivity check. ASA class coded 1 = ASA III, 0 = ASA II (reference). Analysis was performed by linear regression (SPSS Statistics v25.0). * *p* < 0.05.

## Data Availability

The datasets generated and/or analyzed during the current study are available from the corresponding author upon reasonable request.

## Abbreviations

The following abbreviations are used in this manuscript:

CBC	Complete Blood Count
CRP	C-Reactive Protein
ESP	Erector Spinae Plane
IL-1β	Interleukin-1 Beta
IL-2	Interleukin-2
IL-6	Interleukin-6
IL-8	Interleukin-8
IQR	Interquartile Range
LSG	Laparoscopic Sleeve Gastrectomy
MPV	Mean Platelet Volume
NLR	Neutrophil-to-Lymphocyte Ratio
OSAS	Obstructive Sleep Apnea Syndrome
PAI-1	Plasminogen Activator Inhibitor-1
PCT	Plateletcrit
PDW	Platelet Distribution Width
PLR	Platelet-to-Lymphocyte Ratio
PLT	Platelet Count
ROC	Receiver Operating Characteristic
SD	Standard Deviation
SII	Systemic Immune-Inflammation Index
SIRI	Systemic Inflammation Response Index
SPSS	Statistical Package for the Social Sciences
TAP	Transversus Abdominis Plane
TNF-α	Tumor Necrosis Factor Alpha
VAS	Visual Analog Scale
WBC	White Blood Cell Count
